# Minimal memory for details in real life events

**DOI:** 10.1038/s41598-018-33792-2

**Published:** 2018-11-12

**Authors:** Pranav Misra, Alyssa Marconi, Matthew Peterson, Gabriel Kreiman

**Affiliations:** 1 Departments of Ophthalmology Neurology, Children’s Hospital, Harvard Medical School, Pilani, India; 2 Birla Institute of Technology and Science, Pilani, India; 3 Emannuel College, Thiruvanathapuram, Kerala India; 4 Department of Brain and Cognitive Sciences, MIT, Cambridge, USA

## Abstract

The extent to which the details of past experiences are retained or forgotten remains controversial. Some studies suggest massive storage while others describe memories as fallible summary recreations of original events. The discrepancy can be ascribed to the content of memories and how memories are evaluated. Many studies have focused on recalling lists of words/pictures, which lack the critical ingredients of real world memories. Here we quantified the ability to remember details about one hour of real life. We recorded video and eye movements while subjects walked along specified routes and evaluated whether they could distinguish video clips from their own experience from foils. Subjects were minimally above chance in remembering the minutiae of their experiences. Recognition of specific events could be partly explained by a machine-learning model of video contents. These results quantify recognition memory for events in real life and show that the details of everyday experience are largely not retained in memory.

## Introduction

In his famous piece of fiction, “Remembrance of Things Past”, Marcel Proust provocatively suggested that rich details about remote autobiographical events are perpetually stored in our brains and can be retrieved by the right combination of cues. Autobiographical memories constitute the central backbone of our identity^1,2^. Brains are continuously bombarded with complex and rich sensory information, which is filtered, subjectively interpreted and re-encoded. Episodes recalled from memory are relatively short, constructed narratives, with temporal sequences, contextual cues and emotional valence. The extent to which detailed information about the original events is retained or forgotten has been a matter of extensive debate. Furthermore, elucidating which contents are retained has important implications for our understanding of brain representations and for clinical situations that involve memory loss.

Measurements of how much detailed information is retained in episodic memories vary widely. Some studies have argued for massive storage capacity wherein subjects can remember thousands of individual pictures^3^, including detailed information such as specific object poses^4–6^, or even individual frames within movies one year after encoding^7^. However, other studies have demonstrated how labile memories can be^8–10^. The bewildering diversity of answers emphasizes that essentially all aspects of the experimental design in memory studies matter. For example, studies examining whether a picture was presented a few minutes earlier do not imply that the minutia of real world experiences will or will not be remembered; other studies where subjects omit many aspects of an experience during free recall do not imply that those details have been forgotten.

Because of the difficulties inherent in studying naturalistic events, a large body of work on episodic memory has focused on laboratory conditions evaluating how well subjects remember single words, faces, or objects from a list. Real world conditions are strikingly different from experiments based on lists of items since episodic events are embedded in spatial and temporal context, they are colored by emotional valence, they are influenced by current tasks and goals, they are typically encoded in a natural and involuntary fashion, and they depend on the complex and rich interactions of a large number of internal and external variables. Furthermore, behavioral performance under laboratory-based tests may not extrapolate to real-world conditions and even brain areas involved in processing autobiographical memories may be distinct in real world memory tests^11^.

There have been heroic attempts to study episodic memory formation in real life^12^ or approximations to real life, including studies of memories for objects and places (e.g.^13–17^), eyewitness testimonies and “flashbulb” memories (e.g.^18,19^), autobiographical memories from objects or photographs (e.g.^20^), movies (e.g.^7^), naturalistic constructed experiences (e.g.^21,22^), and memory for stories (e.g.^23^, among others. Recently, there has been significant enthusiasm in the use of wearable cameras that can take photographs to document snapshots of real world events^11,24–26^. Wearable cameras provide ground truth information and enhanced ecological validity, and they also hold the potential to enhance memory formation both in healthy individuals as well as in subjects with cognitive impairment.

Quantifying episodic memory formation in the real world is difficult for several reasons: (i) real life events are unique and are not directly amenable to averaging or evaluating repetitions of identical stimuli; (ii) ground truth information is typically hard to attain; (iii) real life events do not have well-defined onsets and offsets; (iv) real life events cannot be controlled in the same way that a list of words can; (v) real life events are mostly not encoded deliberately, as opposed to laboratory experiments where subjects are asked to memorize certain items; (vi) many studies of real life memories are based on subjective evaluation of the answers, in contrast with quantitative tasks more commonly used under laboratory conditions. As a consequence of these challenges, there is still a profound gap between laboratory-based examination of memory for individual items from a list and our understanding of real life memory formation.

Here we quantified detailed recognition memory for real world events by combining video monitoring, eye-tracking and computational models. We monitored and recorded approximately one hour in the life of our subjects while they walked along a specified route, and we subsequently evaluated whether they could distinguish their own personal episodic experiences from those of other subjects that followed the same route on a different day. The results show that subjects were barely able to recognize highly detailed events from their own experience. Part of the subjective filtering criteria selecting which events are to be remembered could be captured by a machine-learning model incorporating information about the visual content of each event.

## Results

**Figure 1:**
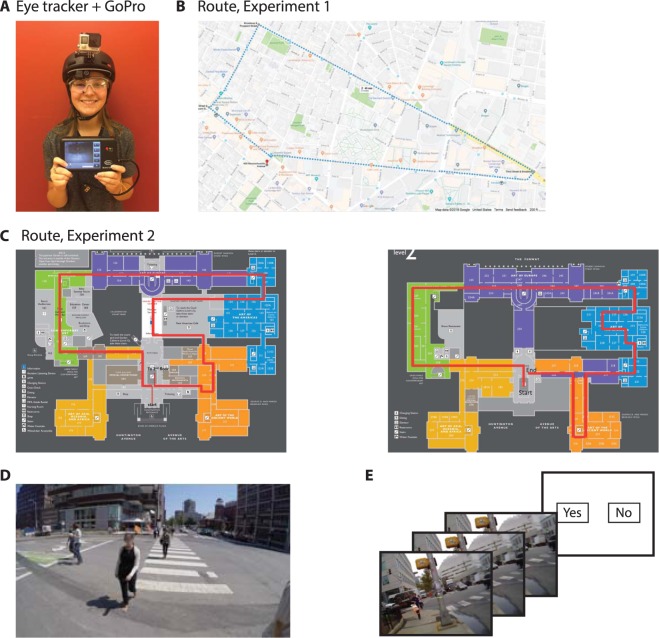
Experiment Design. (**A**) Combined GoPro + Eyelink apparatus to record video and eye movements. (**B**) Route for Experiment I in Cambridge, MA. (**C**) Route for Experiment II along the first floor (left) and second floor (right) of the Boston Museum of Fine Arts. (**D**) Example frame from Experiment I. In this rendering (but not in the actual experiment), all faces were blurred due to copyright issues. (**E**) Recognition memory test. Subjects were presented with one-second video clips and had to indicate whether the video belonged to their own experience or not.

We quantitatively evaluated the extent to which human subjects remember the details of specific events occurring during one hour of their lives. Nineteen subjects participated in either one of two similarly structured experiments. In both experiments, subjects had to walk along a specified route for about one hour (incidental *memory encoding* phase of the experiment, 59 ± 3.4 and 55.4 ± 1.5 minutes (mean ± SD) for Experiments I and II, respectively). To record ground truth information about the events during memory encoding, subjects wore a contraption consisting of an eye tracker and a video camera (Fig. 1A, Methods). The next day, subjects came back to the lab to participate in a memory evaluation test. During this test, subjects were presented with pseudo-randomly interleaved one-second target video clips from their own experience or one-second foil video clips from a different subject that walked along the same route (Figs 1E and S1, Methods). Subjects pressed one key to indicate whether the video clip was part of their own personal experience (Yes) or a different key otherwise (No), in a forced choice manner. The number of target and foil clips was the same; hence chance performance was 50%. There were two experimental variations, with identical structure but different routes (Experiment I, outdoors, Fig. 1B, and Experiment II, indoors, Fig. 1C, Table S1). Three subjects were excluded from the analyses because of concerns about biases in the experimental conditions or responses (Methods).

Figure S1 shows one example target video clip from Experiment 1 (Fig. S1A1) and one from Experiment 2 (Fig. S1B1). Foil video clips were selected from the encoding session of another subject. Figs S1A2 and S1B2 show extreme examples where we selected, for illustration purposes, a foil clip corresponding to approximately the same location (in general, the foils were randomly selected). What distinguishes the personal experience (target) from that of another subject is a combination of what-where-when information about the event. For example, in S1A1, there is one man in a blue shirt walking towards the subject whereas in S1A2 there are 6 people in the video clip, 5 of whom are seen from the back. This experimental structure naturally captures autobiographical episodes to assess whether detailed information about the event is retained (in the same way that a person may take the same route and work in the same location everyday and episodes are distinguished by the combination of people, actions, interactions, and other occurrences, see Discussion).

Unbeknown to the subjects, a small fraction of the video clips was repeated to evaluate self-consistency, i.e., to quantify whether subjects would provide the same response to the same question (Methods). Despite the difficulty of the task, the degree of self-consistency was 78.1 ± 2.9% in Experiment I and 74.9 ± 4.0% in Experiment II (mean ± SEM), both significantly above chance levels (chance = 50%, p < 10^−4^, two-sided t-test). Subjects used “Yes” and “No” responses more or less equally: the proportion of “Yes” responses was 47.2 ± 13.9% in Experiment I and 46.2 ± 10.9% in Experiment II (mean ± SD).

**Figure 2:**
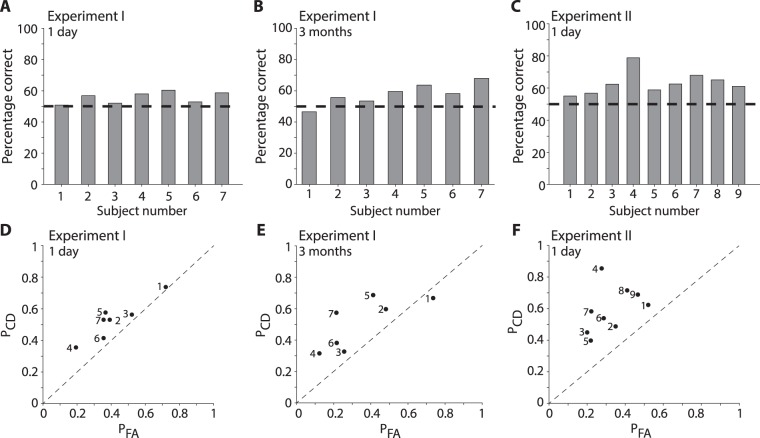
Minimal recognition memory detailed events. (**A**–**C**) Recognition memory task performance for each subject (percentage of correct trials, including correct target detection and correct foil rejection). The horizontal dashed line indicates chance performance. (**D**–**F**) Probability of correct detection (P_CD_) versus the probability of false alarm (P_FA_) for each subject. The diagonal dashed line indicates chance performance. The numbers denote the subject number.

Overall performance in Experiment I was slightly, but significantly, above chance levels: 55.7 ± 3.7% (mean ± SD, p = 0.007, two-sided t-test, Fig. 2A). The number of correct detections (target clips correctly labeled as remembered) was 53% and was comparable to the number of correct rejections (58%, foil clips correctly labeled as not remembered), both of which were above chance (d’ = 0.31 ± 0.19, Fig. S3A). There was considerable variability across subjects in the trade-off between correct detections and false alarms, but all subjects performed slightly above chance levels (Figs 2D and S3A). In Experiment I, subjects were brought back to the lab approximately 3 months after the memory encoding session and were re-tested using the same target clips but different foil clips (Fig. 2B). Subjects were still able to distinguish their own video clips, with a performance of 57.8 ± 7.0% (mean ± SD, p = 0.02, two-sided t-test; d’ = 0.46±0.38, Fig. S3B). The average performance was slightly higher at 3 months compared to 1 day, but this difference was not statistically significant (p > 0.2, two-sided t-test). There was an increased number of correct rejections at 3 months (65%) compared to correct detections (51%) and all except one subject were above chance at 3 months (Figs 2E and S3B). Other investigators have demonstrated a “testing effect” whereby memory performance can remain strong or even increase when the same material is re-tested, even without feedback as is the case for our study^27^.

Similar conclusions were reached for Experiment II, where performance was slightly higher than that in Experiment I. The overall performance in Experiment II was 63.2 ± 7.0% (mean ± SD, p = 0.0005, two-sided t-test, Fig. 2C), with a correct detection rate of 59% and a correct rejection rate of 67% (d’ = 0.72 ± 0.41, Fig. S3C). Individual differences were also apparent in Experiment II, with one subject performing well above the rest (subject 4, Fig. 2C). All subjects performed above chance levels (Figs 2F and S3C).

**Figure 3:**
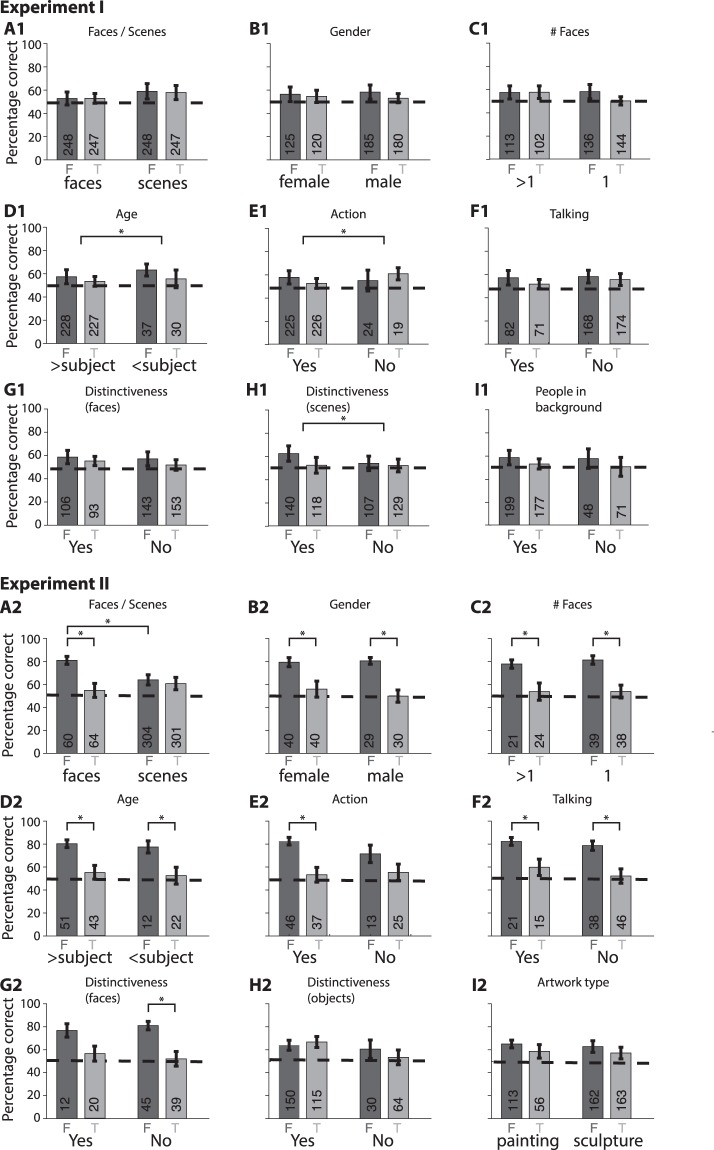
Video clip content and performance for Experiment I (**A1**–**I1**) and Experiment II (**A2**–**I2**). The contents of each video clip were manually annotated based on a list of basic properties (see Methods and Supplementary Material for definitions). Performance is reported separately for foil (F, dark gray) and target (T, light gray) video clips for each content property. For example, **A** separately shows performance for video clips containing faces or video clips containing scenes with no faces. The dashed line indicates chance levels. The * denotes statistical significance (P < 0.01, ranksum test). Error bars indicate SEM. The number of trials for each condition is shown inside each bar.

**Figure 4:**
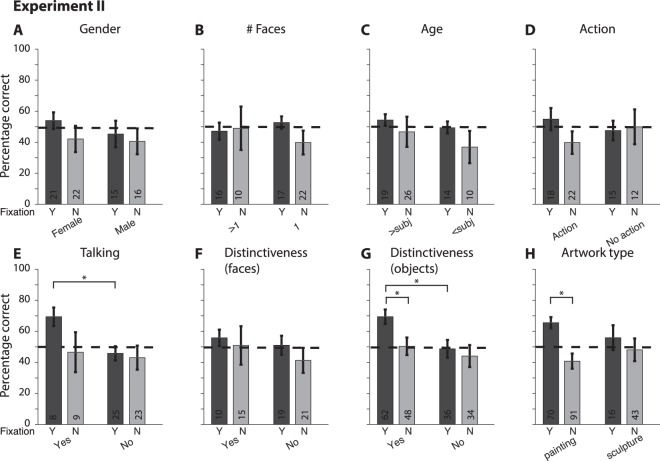
Influence of eye fixations on performance. Fixation targets were manually annotated based on the list of properties from Fig. 3. Performance is reported separately for fixation (Y, dark gray) or no fixation (N, light gray) onto the specific content. For example, **A** separately shows performance for video clips in which there was a female present (left) or a male present (right) and the subject either fixated on the person (Y) or not (N). Only target trials are shown here. Error bars indicate SEM. The * denotes statistical significance (P < 0.01, ranksum test). The number of trials for each condition is shown inside each bar.

**Figure 5:**
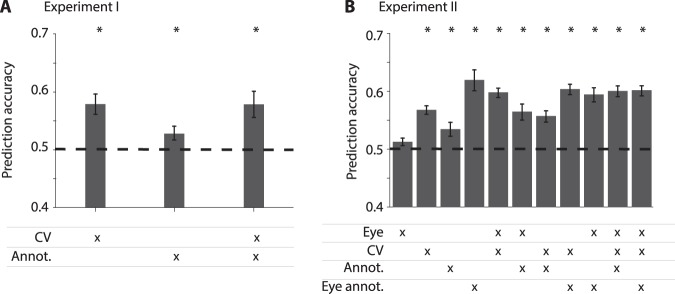
Machine learning performance prediction in single trials. A machine learning model was trained to predict subjects’ performance using a combination of different types of features: computer vision (CV), manual annotations from Fig. 3 (Annot.), eye fixations from the eye tracking data (Eye) and fixation annotations from Fig. 4 (Eye annot.). Details about the machine learning analysis are provided in the Methods section. Chance performance = 0.5. Error bars denote SEM and “*” denotes statistical significance with respect to chance (p < 0.01, t-test).

The decision to evaluate performance in one-second video clips was based on the following criteria and constraints: (i) significantly shorter clips may not have enough information to define actions and events; (ii) significantly longer clips would reduce the temporal resolution and would lead to a large drop in the number of trials (particularly for the subsequent analyses in Figs 3–5); (iii) results from our previous work suggested that one-second clips provide an adequate time frame to evaluate episodic memories^7^; (iv) one-second video clips provide much more information than other studies based on single photographs. It is reasonable to assume that episodic memory events in real life encompass time scales longer than one second^28,29^. We hypothesized that these longer time scales for episodic memory events would be manifested in correlations in performance over scales longer than one second. To test this hypothesis, we computed the conditional probability that performance was correct at time *t* during the memory-encoding phase of the task, given that performance was correct at time *t- Δt* (with *Δt* > 0 providing a temporal scale for the history dependence in episodic memories) (Fig. S4). Under the null hypothesis, performance at a given time point should be independent of performance at other time points and therefore this conditional probability should equal the overall probability of being correct (horizontal dashed line in Fig. S4). For both experiments and both test times in Experiment I, we found that there was a clear correlation in performance spanning multiple seconds. Subjects were more likely to correctly remember a particular event if they had correctly remembered an event occurring a few seconds earlier. We fitted a function of the form $y=1+\alpha {e}^{(-t/\tau )}$ to the curves in Fig. S4, yielding parameters (α = 0.32, τ = 28.4 seconds), (α = 0.375, τ = 37.6 seconds), and (α = 0.13, τ = 10.8 seconds) for the 3 experiments. These results suggest a temporal scale on the order of tens of seconds for the episodic memory events in this study. This temporal scale depends on the rate of movement through the environment and the ensuing correlations in the perceptual details over time, both of which aim to approximate natural conditions in these experiments. The order of the questions during the recognition memory test was pseudo-randomized and there was no correlation in performance at different testing times during the recognition memory test (Fig. S5).

We next sought to better understand what factors contribute to the memorability of a particular video clip (Fig. 3). For this purpose, we combined computer vision and manual annotation of the contents of each video clip. First, we annotated each video clip in terms of a series of pre-defined intuitive variables: presence of faces, gender, number of faces, age of people in the video clip, whether those people were performing any actions, whether they were talking, whether the faces and objects in the clip were distinctive, whether there were people in the background (Experiment I only) and whether there were paintings or sculptures (Experiment II only). Some of these dimensions are clearly subjective; the Supplementary Material provides a description of the criteria used for each of these annotations. All the target and foil video clips were annotated by two of the investigators (P.M. and A.M., Methods). These annotations were performed blindly to the behavioral responses of each subject. Memorable events could contain a variety of contents and performance was largely independent of these annotated variables (Fig. 3), with a few exceptions. In Experiment I, the age of people (Fig. 3D1), whether people were performing actions (Fig. 3E1) and the distinctiveness of scenes (Fig. 3H1), led to a significant difference in performance (p < 0.01, ranksum test). In Experiment II, subjects were more likely to correctly reject having seen a face in the foil trials (Fig. 3A2). In addition, in Experiment II, but not in Experiment I, for most (but not all) of the content annotations, there was a significant difference between targets and foils. Subjects were more likely to correctly reject foil trials (compare dark gray versus light gray bars in Fig. 3A2–I2, these observations are consistent with the results shown in Fig. 2F).

The analyses in Fig. 3 are based on the presence or absence of specific contents in the video clips but it is unclear whether subjects were paying attention to those contents during the memory-encoding phase. Using eye fixations as a coarse proxy for attention, we next sought to investigate whether specific content that was fixated upon was more or less likely to be remembered (Fig. 4). Due to technical difficulties, we could not record accurate eye tracking data during Experiment I (Methods); therefore, this analysis was restricted to Experiment II. Eye movements from other subjects (in foil video clips) are not relevant to this question; therefore, this analysis was restricted only to target trials. Following the procedure in Fig. 3, we annotated the content of each fixation during each video clip, blindly to the subject’s behavioral performance. Subjects showed an enhanced performance when they fixated on a person that was talking (Fig. 4E), on a distinctive object (Fig. 4G), or on a painting (Fig. 4H).

We complemented the features manually annotated in Figs 3–4 with features derived from a computer vision algorithm processing the frames in each video clip (Methods). We used the Alexnet computer vision model, which consists of a 7-layer convolutional network that takes as inputs the pixels of each frame and computes a representation of the input frame in each layer^30^. The representation of images by the Alexnet computational model has been successful in visual classification tasks. Except for the results shown in Fig. S7, we focused on the features from the fc7 layer, that is, the last layer before the one used for object classification, comprising “high-level” features of the visual input. Instead of describing performance separately when a particular video clip feature is present or not as in Figs 3–4, we combined all of the features together to ask whether it is possible to predict whether a particular clip will be correctly remembered or not (Fig. 5). Importantly, this model makes single trial predictions about whether a particular event will be correctly classified as remembered or not. We combined the manual annotations based on the video (Fig. 3), the manual annotations of content in each fixation (Fig. 4), the computer vision annotations and properties of eye movements (number of saccades, saccade duration) within a video clip to build a machine learning classifier model to predict the subjects’ performance. This classifier was trained via cross-validation for each subject and we report the results averaged across subjects in Fig. 5. In Experiment I, a classifier combining computer vision features and manual annotations yielded above chance performance (Fig. 5A, performance = 57.6 ± 1.2%, mean ± SEM, p = 0.007 compared to chance levels, two-sided t-test). This performance was driven mostly by the computer vision annotations. There was a large degree of heterogeneity in the classifier performance for each subject (Fig. S6A), yet in all subjects the classifier performance was above chance. In Experiment II, a classifier combining all features also yielded above chance performance (Fig. 5B, performance = 60.1 ± 0.7%, mean ± SEM, p < 10^−6^ compared to chance levels, ranksum test). In this case, the features that contributed most to the classifier were the manual annotations of content for each fixation (which we could not incorporate into the analyses in Experiment I). As observed in Experiment I, there was variation across subjects, yet the classifier performed above chance levels for all subjects (Fig. S6B).

## Discussion

This study aimed to quantify at high temporal resolution the extent to which detailed episodic information is retained in real life scenarios. Subjects were recruited to walk along a specified route for about one hour while we recorded their first person experiences with a video camera and an eye tracker mounted on their heads (Fig. 1). Subjects subsequently performed an old/new recognition memory task to evaluate whether they remembered events from the encoding phase. We found that memory for one-second events was barely above chance levels (Fig. 2). Some of the event contents (Fig. 3), including their eye fixation contents on a moment-by-moment basis (Fig. 4) could help partly explain and predict (Fig. 5) which events were memorable and which ones were not.

There is extensive discussion in the literature about what exactly is meant by “episodic memory” and what exactly is measured by different memory tasks (for lucid discussions, see^15,31^). The task presented here was based on distinguishing target video clips that reflect personal experiences from foil video clips depicting similar locations and conditions but clearly distinct combinations of what/where/when information. The experimental paradigm strived to map onto a real world scenario, such as a person who takes the same route to work every day, yet several potential caveats merit discussion. Neither the recruitment nor the task instructions included any specific reference to “memory”, in an effort to ensure the incidental nature of memory formation. Yet, it is conceivable that some subjects may have surmised a connection to memory experiments given that they knew ahead of time that they would return to the lab the next day, and also potentially by searching background information online on the lab’s website. To the best of our knowledge, and based on subjective personal reports, subjects did not deliberately attempt to “rehearse” or “recall” the events that happened during the encoding session before the test session. Although there was no reward or motivation for such rehearsals or recollections, we cannot exclude the possibility that subjects may have attempted to do so. To the extent that they did, the current results would represent an overestimate of the degree of episodic memory formation for detailed information during real life conditions. Even though the stated goal was to examine situations that were as natural as possible, the experimental setup required several compromises: (i) subjects had to wear a contraption including a helmet, a video camera and an eye tracker; (ii) subjects were paid to participate in the task; (iii) subjects had to idly walk along a specified path for one hour; (iv) subjects had to recalibrate the eye tracker repeatedly; (v) subjects were aware that they had to come back to the lab for another test; (vi) subjects were followed by one of the investigators. All of these factors combine to make the task less natural than true real life scenarios. If anything, we speculate that these unusual circumstances might contribute to enhancing memory formation (because it may be more memorable to be paid to walk for one hour with a camera and an eye-tracker on your head). Due to subjects potentially inferring a connection to memory, subjects potentially rehearsing information after the encoding session and the non-natural aspects of the task, the minimally above chance performance reported here may actually constitute an overestimate of detailed memory formation in real life.

The type of material presented during the encoding phase and how the material is acquired has a large influence on performance. To a reasonably good approximation, subjects in our task formed memories spontaneously, as opposed to studies in which subjects are instructed or otherwise encouraged to deliberately remember certain items. Intention can play an important role in memory formation^15,32,33^. Subjects were actively navigating the environment during our task, as opposed to tasks that involve exclusively passive exposure to incoming information^32^.

Some of the visual contents of each frame correlated with performance. The features that were most correlated with performance included the presence of people and their ages, whether they were performing any actions as opposed to being static, and a subjective notion of distinctiveness (Fig. 3). These annotated content features, combined with computer vision features could be used to partly predict whether subjects would be correct in detecting the target or rejecting a foil (Figs 5 and S6–S7, see also^7^). The machine learning models were barely, but significantly, above chance in predicting recognition memory both at the group level as well as at the individual subject level. We speculate that future and improved versions of this type of models might find interesting applications to predict memorability in real life situations such as what is memorable in a speech, in a class, in a movie, in advertisements, or other real life events.

What subjects fixated on also correlated with performance (Fig. 4), but it is clear that subjects did not remember everything that they laid eyes on. There is extensive literature showing that subjects may not pay attention or be conscious of what they are fixating on (e.g.^34^). Therefore, it is quite likely that, in several instances, subjects may have fixated on an object without necessarily paying attention to that object. Additionally, attention is correlated with the encoding of events into memory. Thus, the current results are consistent with the notion that eye fixations correlate with episodic memory but they are neither necessary nor sufficient for successful episodic memory formation.

In addition to the content of each video clip, it is likely that its duration has an impact on performance. In this study, the duration of the video clips during the recognition memory test was fixed at 1 second. In general, events in real life have temporal scales spanning several seconds up to several tends of seconds^28,29^. Consistent with those characteristic event durations, we found that recognition memory was correlated on scales of several seconds (Fig. S4). Furthermore, previous work using movies to evaluate episodic memories showed a small but significant increase in performance with video clip duration^7^. It is likely that longer video clips would lead to higher performance, but the focus of this study was in achieving high temporal resolution and to be able to separate contents across video clips.

Even basic visual features automatically calculated by a computer vision algorithm could be used to partly predict performance (Fig. 5, see also^7,35^). In a previous experiment with a similar structure, subjects watched a commercial movie during the encoding phase and the foils were carefully selected to match the target video clips^7^. In that study, the percentage of correctly remembered events one day post-encoding was above 80%, which is well above the results presented in Fig. 2. There are many differences between a commercial movie 7 and one hour of walking in Experiments 1 and 2 (current study). The movie has a narrative structure, dictated by a director that manipulates attention to entertain the viewer, with a large number of events with high emotional valence that are temporally compressed, and a much larger variety of different locations and distinct events.

In addition to the nature of material presented during encoding, the way in which memories are tested is also critical to interpreting the results. In the type of old/new forced choice tasks such as the one used in the current study, the nature of the foil trials plays a critical role in performance. The task can be made arbitrarily easier or harder by choosing different foils (e.g. if the foil video clips are identical to the target video clips except that the image is mirror reflected, the task becomes extremely hard^7^, whereas if the foil video clips were taken from a completely different video sequence, the task would be extremely easy).

A forced choice old/new mechanism was used to scrutinize details about memories at a resolution of one-second in a recognition memory test that involved discriminating whether a video clip was part of the subjects’ own personal experience or not. This evaluation mechanism is different from other studies that have focused on free recall, diaries or questionnaires. It should be emphasized that the focus here is on whether the details of everyday experience are retained and the results should not be interpreted to imply that all memories are lost. As expected, and based on subjective reports after the test, subjects could still recall that they had taken part in the encoding phase and could also spontaneously provide information about the encoding phase such as where they walked, the time of day, the equipment they used, etc. Thus, the current results should *not* be taken to imply that subjects had completely forgotten everything that happened during the encoding phase of the task. As demonstrated in previous work, the current results are consistent with the idea that the events occurring during encoding are filtered, synthesized, and re-structured into a compressed narrative. What the current results show is that only minimal details are retained.

It can be surmised that subjects could recur to educated guessing as part of their strategy during the task. We strived to minimize the differences between target and foil video clips but this was not always possible. An extreme case happened in one of the subjects in Experiment I for whom the weather conditions were significantly different than the rest. In this case, recalling only one bit of information (weather conditions) was sufficient for the subject to distinguish his own video clips at 91% accuracy. While recalling the weather is still an aspect of real life memory, this was not informative regarding the ability to form detailed memories for each individual event and therefore this subject was excluded from the analyses. Even though it was generally quite difficult to differentiate target and foil video clips (see examples in Fig. S1), more subtle versions of educated guessing, which are largely but not entirely independent of detailed episodic memory formation, could have taken place during the test. Such educated guessing could lead to overestimating performance, further reinforcing the conclusions that only minimal aspects of the details of daily experience are remembered.

The methodology introduced in this study fulfills six of the seven criteria stipulated by Pause and colleagues for a valid measure of episodic memory^31^: there was no explicit instruction to memorize any material, events were either of neutral valence or contained everyday emotional valence, memory encoding was induced in single trials, the episodic information contained what/where/when information in a natural setting, the memory test was approximately unexpected and the retention interval was over 60 minutes. The only criterion not fulfilled here is that memories were induced in the real world as opposed to laboratory conditions.

While the current work aims to examine “real life” events, it is clear that the task only scratches the surface of what we ultimately need to understand. Real life is much more dynamic and interesting than following a pre-specified route: we have experiences relevant to our goals, history, identity and relationships, we make decisions about which way to go or when to stop. Such self-relevant details can be more salient than a stranger or a car moving past. Moreover, external experiences are accompanied by ongoing inner experience that often captures our attention at the expense of external stimuli. These and many other aspects of real life experiences are accounted for by the current task. A full understanding of episodic memory formation for real life events will certainly have to include the connections between sensory events and internal experiences, goals, personal history and social interactions.

Understanding and measuring episodic memory formation has important implications for assessing, and eventually helping, clinical populations with cognitive deficits^14,31,36–39^. Deterioration in episodic memory formation is a critical hallmark of conditions such as cognitive dementia. There has been notable progress in uncovering the brain circuits responsible for episodic memory formation (e.g.^11,24,25,40–43^, among many others). A more accurate and systematic account of episodic memory at the behavioral and theoretical level can play a crucial role in better dissecting the distinct contributions of different neural circuits to memory formation and also in better understanding the consequences of malfunction in those circuits.

There has been considerable discussion about the extent to which information is irretrievably lost during the encoding phase or whether a large fraction, if not all, information is actually stored but there is a subsequent inability to retrieve the information^4,7–10,40,44–46^. Under the assumption that the video-clip presentations represent an adequate, perhaps even powerful, way of probing recognition memory, the current results strongly suggest that there is significant loss of detailed information at the moment of encoding or in between the encoding phase and the test session. We conclude that most of the moment-by-moment minutiae of daily experience are not crystallized in the form of episodic memories.

## Methods

An expanded version of the Methods can be found in the Supplementary Material.

### Subjects

A total of 19 subjects (18–22 years old, 11 female) participated in these experiments. The target number of subjects was defined before data collection based on the results of one of our previous studies which had a similar structure^7^.

All experimental protocols were approved by the Institutional Review Board at Children’s Hospital and Massachusetts Institute of Technology. All methods were carried out in accordance with the approved guidelines. Written informed consent was obtained from all subjects. Figure 1A shows a picture of one of our subjects wearing the device used to capture video and eye tracking movements; the subject provided informed consent for publication of her image in an online open-access publication.

### Memory encoding

The overall structure of the task was similar to that in previous studies^7,26,43^. In the first phase of the protocol (memory encoding), each subject had to walk along a pre-specified route (Fig. 1B,C). In the second phase of the protocol (memory evaluation), subjects came back to the lab to perform a memory task (Fig. 1E, described below). There were two experiment variants.

### Experiment I

Subjects were instructed to follow a specified and fixed 2.1-mile route in Cambridge, MA (Fig. 1B). Subjects were relatively unfamiliar with the area: most of the route was unfamiliar but some of the streets may have been visited on a few occasions before. Subjects spent 59 ± 3.4 minutes (mean ± SD) on this route. The experimenter (A.M.) walked behind the subject to ensure that the equipment was working properly and that the subject was following the specified route.

### Experiment II

The format of the experiment was similar to Experiment I. In Experiment II, the route was indoors in order to increase the accuracy of the eye tracking measurements (see below). Subjects were instructed to follow a specified and fixed path within the Museum of Fine Arts (MFA) in Boston (Fig. 1C). Subjects were unfamiliar with the MFA before the experiment (they had not visited the MFA before). Subjects spent 55.4 ± 1.5 minutes (mean ± SD) on this route.

### Video recordings and eye tracking

A Mobil Eye XG unit (ASL Eye Tracking, Bedford, MA) was fitted on the subject along with a GoPro Hero 4 Silver camera (GoPro, San Mateo, CA, Fig. 1A). Details about the initial calibration of the eye tracker, subsequent recalibrations, fixation detections, and alignment between the GoPro video and eye tracking signal are provided in the Supplementary Material.

### Memory evaluation

Subjects came back to the lab one day (24 to 30 hours) after the memory-encoding phase of the experiment. Memory evaluation was based on a recognition memory test following essentially the same protocol that we published previously when studying memory for movie events^7^. After presentation of each one-second video clip, subjects performed an old/new task where they had to respond in a forced choice manner indicating whether or not they remembered the video clip as part of their own experience during the memory-encoding phase (Fig. 1E). Subjects were presented with an equal proportion of targets (video clip segments taken from their own memory encoding sessions) and foils (video clip segments taken from another subject’s memory encoding session). Target or foil clips were shown in pseudo-random order with equal probability (see Supplementary Fig. S8 for post-hoc power analyses for the number of trials). In Experiment I, subjects were also asked to come back to complete an additional memory evaluation test three months after the memory encoding phase. Target and foil clips were selected from the set of videos recorded during the memory-encoding phase (Fig. S1). The average interval between clips was 7.07 ± 0.89 seconds and 7.50 ± 0.32 seconds in Experiment I and Experiment II, respectively (Fig. S2, trial order was pseudo-randomized). Additionally, a total of 50 clips for Experiment I (25 target clips and 25 foil clips) and 36 clips for Experiment II (18 target clips and 18 foil clips) were repeated to evaluate self-consistency in the behavioral responses (unbeknown to the subjects). The degree of self-consistency was 78.1 ± 2.9% and 74.9 ± 4.0% (mean ± SEM) for Experiment I and Experiment II, respectively (where chance would be 50% if the subjects responded randomly). The number of foil clips matched the number of target clips such that chance performance in the recognition memory task was 50%. All video clips were pseudo-randomly interleaved. Subjects were not provided with any feedback regarding their performance. Examples of frames from target and foil clips are shown in Fig. S1.

### Data analyses

Two subjects from Experiment I were excluded from the analyses. One of these subjects had a score of 96%, which was well above the performance of any of the other subjects (Fig. 2). The weather conditions on the day of the walk for this subject were substantially different, and this subject could thus easily recognize his own video clips purely from assessing the weather conditions. Another subject was excluded because he responded “yes” >90% of the trials.

### Video clip content properties

To evaluate what factors determine the efficacy of episodic memory formation, we examined the content of the video clips by using computer vision models and manual annotations. Video clips were manually annotated by two of the authors (A.M. and P.M.). These annotations were performed blindly to the subjects’ behavioral responses during the recognition memory test. In the few cases of inter-rater disagreement (<15%), the annotations by P.M. were used. Table S2 provides a brief definition for each of the annotations used in Figs 3–5. In Experiment II, in addition to the contents of each video clip we also examined whether the characteristics of eye fixations were correlated with episodic memory formation. For this purpose, we re-evaluated the content properties based on what subjects fixated upon. Only target trials were analyzed in Fig. 4 because foil trials come from a different subject and the pattern of fixations of a *different* subject is not directly relevant for a given subject’s performance in the recognition memory task.

### Predicting memorability

We developed a machine-learning model to evaluate whether it is possible to predict memorability for individual video clips based on the contents of each clip and eye movement data. We trained a classifier to learn the map between those content features and the video clip memorability following the methodology described in^7^, see also machine learning approaches applied to memorability for images^35^. We used cross-validation and decision trees with the Adaboost algorithm as a classifier (qualitatively similar results were obtained using a support vector machine classifier with an RBF kernel).

## Electronic supplementary material

Supplementary Material

## Data Availability

All the data and open-source codes used for this study will be made publicly available upon acceptance of the manuscript via the authors’ website: http://klab.tch.harvard.edu.
