# Supplementary material for: Minimal memory for details in real life events
Source: Sci Rep. 2018 Nov 12;8:16701. doi: 10.1038/s41598-018-33792-2 (PMC6232115; doi:10.1038/s41598-018-33792-2)
Supplement: Supplementary file 1 — Supplementary Material [file 41598_2018_33792_MOESM1_ESM.pdf]

Supplementary Material

Minimal memory for details in real life events

Pranav Misra[1,2], Alyssa Marconi[1,3], Matthew Peterson[4], Gabriel Kreiman[1*]

[1]Departments of Ophthalmology and Neurosurgery, Children's Hospital, Harvard Medical School
[2]Birla Institute of Technology and Science, Pilani, India.
[3]Emannuel College
[4]Department of Brain and Cognitive Science, MIT

[*]To whom correspondence should be addressed: gabriel.kreiman@tch.harvard.edu

1. **Supplementary Methods**
2. **Supplementary Tables**
3. **Supplementary Figures**

1. Supplementary Methods

Subjects

A total of 19 subjects participated in these experiments. All of the subjects were college students between 18 and 22 years old. As described below, there were two experiment variants: 9 subjects (5 female) participated in Experiment I and 10 subjects (6 female) participated in Experiment II. Subjects received monetary compensation for their participation in the study. All experimental protocols were approved by the Institutional Review Board at Children's Hospital and Massachusetts Institute of Technology. All methods were carried out in accordance with the approved guidelines. Informed consent was obtained from all subjects.

Memory encoding

Subjects were recruited to participate in a protocol “assessing everyday, natural visual experience”. The recruitment and task instructions did not include any mention of “memory” studies. The overall structure of the task was similar to that in previous studies (St Jacques and Schacter, 2013; Dede et al., 2016; Tang et al., 2016). In the first phase of the protocol (memory encoding), each subject had to walk along a pre-specified route (**Figure 1B-C**). In the second phase of the protocol (memory evaluation), subjects came back to the lab to perform a memory task (**Figure 1E**, described below). During the memory-encoding phase, subjects were not given any task; the instructions were to walk along the assigned route with the video and eye tracking apparatus (**Figure 1A**).

There were two experiment variants.

Experiment I. Subjects were instructed to follow a specified and fixed 2.1-mile route in Cambridge, MA (**Figure 1B**). During the preliminary evaluation, we estimated that that it would take about 55 minutes to complete this route. Subjects spent 59±3.4 minutes (mean±SD) on this route. The experimenter (A.M.) walked behind the subject to ensure that the equipment was working properly and that the subject was following the specified route. The route was designed to minimize the number of turns. There were 3 right turns (**Figure 1B**), the first one was very clear because the street ended at that intersection. Thus, there was a maximum of 2 interruptions to provide directions. Each subject participated in the memory-encoding phase on different weekdays. Several measures were implemented in an attempt to maximize the degree of between-subject consistency in the physical properties and subjects’ knowledge / familiarity with the environment: (i) all experiments were run during the course of two summer months (July/August); (ii) experiments were only conducted if the weather conditions were approximately similar (i.e. we avoided rainy conditions or cloudy days since these could provide additional global external cues, see discussion in the main text); (iii) all subjects started at approximately the same time of day (between 12pm and 2pm); (iv) all subjects were students attending Emmanuel College (about three miles away from the specified route) and were not particularly familiar with the specified route before the beginning of the experiment.

Experiment II. The format of the experiment was similar to Experiment I. In Experiment II, the route was indoors in order to increase the accuracy of the eye tracking measurements (see below). Subjects were instructed to follow a specified and fixed path within the Museum of Fine Arts (MFA) in Boston (**Figure 1C**). In the preliminary tests, we estimated that it would take about 50 minutes to complete this route. Subjects spent 55.4±1.5 minutes (mean±SD) on this route. The experimenter (A.M.) accompanied the subjects to ensure that the equipment was working properly and that the subject was following the specified route. In addition, the Museum required that an additional Museum intern accompany the subject during the entire test. The routes were designed to minimize the number of turns. Subjects had to continue walking straight, and they were never to go back to the same museum rooms that had already been visited. There was a total of 12 turns that were not obviously specified by these two instructions. Subjects performed the test on different weekdays. Several measures were implemented in an attempt to maximize the degree of between-subject consistency in the physical properties and subjects' knowledge / familiarity with the environment: (i) all experiments were run during the course of two winter months (January/February); (ii) all subjects started at approximately the same time of day (between 12pm and 2pm); (iii) all subjects were college students attending Emmanuel College and were not familiar with the Museum. There was no overlap between the subjects that participated in Experiments I and II.

Video recordings and eye tracking

Apparatus. A Mobil Eye XG unit (ASL Eye Tracking, Bedford, MA) was fitted on the subject along with a GoPro Hero 4 Silver camera (GoPro, San Mateo, CA). The setup is shown in **Figure 1A**. The Applied Science Laboratory (ASL) Mobile Eye-XG Tracking Glasses measure real-world gaze direction at 60 samples per second. The ASL glasses utilize two cameras: a scene camera and an eye camera. The scene camera sits on top of the rim of the glasses (**Figure 1A**). The camera was adjusted for each subject to align the center of the camera's field of view (FOV) with the center of the subject. The

scene camera FOV spanned 64° horizontally and 48° vertically with a resolution of 640 by 480 pixels. To estimate gaze direction, the eye camera records an infrared (IR) image of the subject's right eye. The IR image contains two sources of information for inferring gaze: the center of the pupil and the position of a pattern of three IR dots from an IR emitter that reflects off the cornea. The eye camera was adjusted so the three reflected dots were centered onto the subject's pupil. To improve the ASL scene camera's field of view, video quality, and resolution, a GoPro Hero 4 Silver camera was used, recording at 30 fps with a resolution of 2704 by 2028 pixels and a FOV spanning 110° horizontally and 90° vertically (Peterson et al., 2016). The GoPro camera was mounted on the center of a Giro bike helmet using a GoPro front helmet mount. The GoPro camera was mounted on the center of the helmet, which was positioned 3.5 inches (y-direction) above the scene camera and 0.5 inches (xdirection) to the right (**Figure 1A**). The GoPro camera has a fish-eye distortion; therefore, the fixations analyzed when the two cameras were synchronized focused within the subject's central region (Peterson et al., 2016).

Initial Calibration. Once the GoPro camera and eye tracker were properly fitted, the subject completed a standardized calibration task implemented in the Psychophysics Toolbox 3.0.10 (Brainard, 1997) written in MATLAB (Mathworks, Natick, MA) on a 13" MacBook Pro laptop (Apple, Cupertino, CA). Subjects were first asked to fixate on a centrally presented black dot that contained a white circular center for 2 seconds. After initial fixation, the same dot moved every 2 seconds through a sequence of 12 other positions arranged in a 4 x 3 grid space on the screen in pseudo-random order. Once all 13 dots (12 positions + center fixation) were fixated upon, the entire array of dots appeared and subjects were asked to look again at each dot starting at the upper left corner and moving across each row. The random dot sequence data were used to calibrate the ASL eye tracker using ASL's Eye XG software. In this process, a rater viewed the scene camera footage at 8 fps with the pupil and corneal reflection data from the eye camera overlaid. For each dot transition, the rater waited until the subject moved and stabilized their gaze on the new dot location, ascertained by an abrupt shift in the overlaid pupil and corneal reflection data, and used a mouse to click on the center of dot in the

scene camera image. Once the subject fixated on a new dot, the cursor was moved, and this was continued for the duration of the calibration. The ASL Eye XG software computes a function which maps the displacement vector (pupil center to IR dot pattern) from the eye camera to the pixel coordinates of the dot locations of the scene camera for each of the 13 calibration dots (Peterson et al., 2016). The subsequent dot array data were used to validate the initial calibration and estimate error.

Fixation detection. During the actual experiment, the ASL Eye XG software used the mapping function computed from calibration to calculate and record the subject's gaze location relative to the scene camera image. Frames that included blinks or extreme external IR illuminations (which precluded measurement of the corneal reflection) were excluded from analyses. A “fixation” was defined by the ASL software’s algorithm as an event where there were six or more consecutive samples that were within one degree.

Synchronization of the ASL Eye Tracker and GoPro. To sync the video footage from the ASL eye tracker to the HD GoPro footage a 12x7 checkerboard pattern was presented on the monitor during initial calibration. An automated synchronization script searched for the first frame in the eye tracker scene camera and the GoPro footage when the checkerboard was first detected and synchronized the videos by aligning the checkerboard onset times. From this alignment, a projective linear transform matrix was used to map the 192 vertex points from the ASL to the GoPro’s coordinates. This matrix was used to map gaze coordinates for each frame and each fixation event from the ASL to the GoPro videos (Peterson et al., 2016).

Recalibration of Eye-tracker and GoPro Camera. To validate the subjects’ gaze coordinates throughout the encoding portion of their study, recalibration was regularly performed every 5 minutes (Experiment I) or every 10 minutes (Experiment II). During each recalibration event, the subject held a 12x7 checkerboard at arm’s length and centered at eye level. Subjects were instructed to fixate for two seconds each at the upper left (labeled “1”), upper right (labeled “2”), lower left (labeled “3”), lower right (labeled “4”), and the center (labeled “5”) squares of the checkerboard. Post hoc, the same

calibration procedure described above was used on each recalibration to correct for any drifts or other displacements from the previous calibration.

Analysis of eye tracking data. Despite our efforts, we were unable to obtain high-quality eye tracking data during Experiment I. The main challenge seems to be that the experiments were conducted outside during the daytime in summer, where the large amount of high-intensity infrared light from the bright, diffuse sunlight overwhelmed the visibility of the pupil and corneal reflection in the eye camera's IR image. Due to the lack of consistency and the small segments of high-reliability eye tracking data, we decided to exclude the eye tracking data during Experiment I from the analyses. In contrast, we were able to secure high quality eye-tracking information during Experiment II, which was conducted indoors under ideal, low IR lighting conditions, and the analyses of these data are described below.

Memory evaluation

Subjects came back to the lab one day (24 to 30 hours) after the memory-encoding phase of the experiment. Memory evaluation was based on a recognition memory test following essentially the same protocol that we published previously when studying memory for movie events (Tang et al., 2016). All but two subjects were presented with 1,050 one-second video clips (Experiment I) or 736 one-second video clips (Experiment II). For one subject in Experiment I, the GoPro camera was off-centered during part of the route and we ended up using only a total of 630 video clips. For another subject in Experiment II, the GoPro camera turned itself off, losing video tracking of the last part of the route, and we ended up using only 672 video clips.

After presentation of each one-second video clip, subjects performed an old/new task where they had to respond in a forced choice manner indicating whether or not they remembered the video clip as part of their own experience during the memory-encoding phase (**Figure 1E**). All the video clips were shown at 30 fps, subtending 15 degrees of visual angle. Subjects were presented with an equal proportion of targets (video clip segments taken from their own memory encoding sessions) and foils (video clip segments taken from another subject's memory encoding session). Target or foil clips were shown

in pseudo-random order with equal probability. In Experiment I, subjects were also asked to come back to complete an additional memory evaluation test three months after the memory encoding phase. This second test session followed the same format as the first one. In this second session, the target clips remained the same but the foil clips were different from the ones in the first test session.

Target and foil clips were selected from the set of videos recorded during the memory-encoding phase (**Figure S1, Supplementary Videos 1**). In Experiment I, there were 500 target clips and 500 foil clips. In Experiment II, there were 375 target clips and 375 foil clips. These clips were selected approximately uniformly from the entire encoding phase. The average interval between clips was 7.07±0.89 seconds and 7.50±0.32 seconds in Experiment I and Experiment II, respectively (**Figure S2**, trial order was pseudo-randomized, this figure takes the minimum temporal difference between test clips based on their mapping onto the encoding phase and plots the distribution of those temporal differences). Additionally, a total of 50 clips for Experiment I (25 target clips and 25 foil clips) and 36 clips for Experiment II (18 target clips and 18 foil clips) were repeated to evaluate self-consistency in the behavioral responses (unbeknown to the subjects). The degree of self-consistency was 78.1±2.9% and 74.9±4.0% (mean±SEM) for Experiment I and Experiment II, respectively (where chance would be 50% if the subjects responded randomly).

Each one-second clip was visually inspected for presence of faces. “Face clips” included a person's face (any person) within the one-second clip. “Scene clips” were defined as videos that did not have a person’s face directly within the field of view. Scene clips could still include far away people or people in the background. In Experiment I, half of the trials in the recognition memory test included face clips and the other half included scene clips. In Experiment II, ~15% of the clips contained face clips while the remaining clips included scenes of the various artwork that the subjects examined during the memory encoding phase.

In addition to the encoding content, how memories are tested is critical to interpreting the results. In old/new forced choice tasks, the nature of the foil trials plays a critical role in performance. The task can be made arbitrarily easier or harder by choosing different foils (e.g. if the foil frames are mirror reflections of the target frames, the task

becomes extremely hard (Tang et al., 2016), whereas if the foil frames come from a completely different video sequence, the task becomes extremely easy). The foil clips were taken from a different control subject who walked the same route, at the same time of day, under similar weather conditions, but on a different day. The idea was to mimic real life conditions such as a scenario where a person may commute to work along the same route every day. Foil clips were taken from all sections of the entire route, as were the subject's target clips. Foil clips included the same proportion of face clips described in the previous paragraph. These selection criteria for foil clips allowed for a natural comparison between targets and foils. We used two sets of foil clips, one for the first half of the subjects, and another one for the second half, to account for potential weekly variations in weather, clothing, or any potential inherent biases in the selection of the foil clips. The number of foil clips matched the number of target clips such that chance performance in the recognition memory task was 50%. All video clips were pseudo-randomly interleaved. Subjects were not provided with any feedback regarding their performance. Examples of frames from target and foil clips are shown in **Figure S1** and example video clips are shown in **Supplementary Video 1**.

Subjects could recur to educated guessing as part of their strategy during the task. We strived to minimize the differences between target and foil video clips but this was not always possible. An extreme case happened in one subject in Experiment I where the weather conditions were different than the rest: recalling only one bit of information (weather) was sufficient for the subject to distinguish his own video clips at 91% accuracy. While recalling the weather is still an aspect of memory, this was not informative regarding the ability to form detailed memories for each event and this subject was excluded from the analyses. Perceptually differentiating target and foil video clips was quite challenging (see examples in **Figure S1** and **Supplementary Video 1**), yet subtle versions of educated guessing, which are largely but not entirely independent of memory, could take place during the test. Such educated guessing could lead to overestimating performance, further reinforcing the conclusions that only minimal aspects of the details of daily experience are remembered.

The methodology introduced in this study fulfills six of the seven criteria stipulated by Pause and colleagues for a valid measure of episodic memory (Pause et al.,

2013): no explicit instruction to memorize any material, events containing natural emotional valence, memory encoding induced in single trials, episodic information containing natural what/where/when information, approximately unexpected memory test, and retention interval over 60 minutes. The only criterion not fulfilled here is that memories were induced in the real world as opposed to laboratory conditions.

Data analyses

Data preprocessing. Two subjects from Experiment I were excluded from the analyses. One of these subjects had a score of 96%, which was well above the performance of any of the other subjects (**Figure 2**). The weather conditions on the day of the walk for this subject were substantially different, and this subject could thus easily recognize his own video clips purely from assessing the weather conditions. Another subject was excluded because he responded “yes” >90% of the trials.

Performance. Performance was summarized by computing the overall percentage of trials where subjects were correct. The overall percentage correct includes the number of target clips where the subject responded “yes” (correct detection) and the number of foil clips where the subject responded “no” (correct rejection) (Tang et al., 2016). Additionally, **Figure 2** shows the proportion of correct detections as a function of the proportion of false alarms and **Figure 3** separately shows performance for target clips and foil clips.

Video clip content properties. To evaluate what factors determine the efficacy of episodic memory formation, we examined the content of the video clips by using computer vision models and manual annotations. Video clips were manually annotated by two of the authors (A.M. and P.M.). These annotations were performed blindly to the subjects’ behavioral responses during the recognition memory test. The Supplementary Material provides a brief definition for each of the annotations used in **Figures 3-5**. In Experiment II, in addition to the contents of each video clip we also examined whether the characteristics of eye fixations were correlated with episodic memory formation. For this purpose, we re-evaluated the content

properties based on what subjects fixated upon. For example, for the gender property, we considered the following four possible annotations: "Female Fixation" (i.e., a female face was present in the video clip and the subject fixated on that face), "Female No Fixation" (i.e., a female face was present in the video clip but the subject did not fixate on that face), and similarly, "Male Fixation", "Male No Fixation". Only target trials were analyzed in **Figure 4** because foil trials come from a different subject and the pattern of fixations of a *different* subject is not directly relevant for a given subject's performance in the recognition memory task.

Predicting memorability. We developed a machine learning model to evaluate whether it is possible to predict memorability for individual video clips based on the contents of each clip and eye movement data. Briefly, each video clip is associated with a series of content properties (as defined in the previous section, see also Supplementary Materials) as well as information about eye positions; we train a classifier to learn the map between those features and the memorability of the video clip. The approach follows the methodology described in (Tang et al., 2016).

We used the following content properties:

(i) *Annotations* (labeled *"Annot"* in **Figure 5**). These are manual annotations defined above ("*Video clip content properties"*) and in the Supplementary Materials: presence of faces, gender, age, number of people in the video clip, actions, person distinctiveness, talking, interactions, other movement, non-person distinctiveness, and presence of artwork in Experiment II.

(ii) *Computer vision features* (labeled *"CV"* in **Figure 5**). For each one-second video clip, we considered five frames, uniformly spaced from the first to the last frame in the video clip, and used a computer vision model called Alexnet (Krizhevsky et al., 2012) to extract visual features from the frames. Briefly, Alexnet consists of a deep convolutional network architecture that contains eight layers with a concatenation of linear and non-linear steps that build progressively more complex and transformation-invariant features. Each frame was resized to 227x227 pixels, and we used an Alexnet implementation pre-trained for object classification using the Imagenet 2012 data set. In the main text (**Figure 5**), we focused on the features in the "fc7" layer, the last layer before the object

classification layer; **Figure S7** shows results based on using only pixel information or using other Alexnet layers.

(iii) *Eye tracking data* (used only for Experiment II, labeled *"Eye"* in **Figure 5**). This comprised a vector with three values: the average duration of fixations during the one-second video clip, and the average magnitude of the saccades in the horizontal and vertical axes during the one-second clip.

(iv) *Eye fixation annotations* (labeled *"Eye Annot"* in **Figure 5**). These are manual annotations of the content of each eye fixation (described in "*Video clip content properties"* and Supplementary Materials). The distinction between (ii) and (iv) is that (ii) (*Annot*) refers to the overall contents in the video clip whereas (iv) (*Eye Annot*) specifically refers to what the subject was looking at.

We considered the four types of features jointly or separately in the analyses shown in **Figure 5** and **Figure S6**. Each video clip was associated with a performance label that indicated whether the subject's response was correct or not. A correct response could correspond to a target video clip where the subject responded "yes" or a foil video clip where the subject responded "no". Conversely, an incorrect response could correspond to a target video clip where the subject responded "no" or a foil video clip where the subject responded "yes". Thus, the aim of the classifier was to predict in single trials whether a subject could correctly identify a clip as a target or a foil and therefore correctly remember his/her own experience as distinct from somebody else's video clips. We sub-sampled the number of video clips by randomly selecting the maximum equal possible number of target and foil clips such that chance performance for the classifier was 50%. In the case of Experiment II and in those analyses that involved using the eye tracking data, only target video clips were used (since the eye tracking data from foil video clips belonged to a different subject and we do not expect that a given subject's memorability could be influenced by the pattern of eye movements in a different subject). We still subsampled the correct and incorrect trials such that chance performance was 50%.

We used cross-validation by separating the data into a training set (3/4 of the data) and an independent test set (1/4 of the data). We used an ensemble of 15 decision trees with the Adaboost algorithm as a classifier (qualitatively similar results were obtained

using a support vector machine classifier with an RBF kernel). The results presented in the text correspond to the average over 100 random cross-validation splits.

Data availability

All the data and open-source codes used for this study will be made publicly available upon acceptance of the manuscript via the authors' website: http://klab.tch.harvard.edu

2. Supplementary Tables

Table S1: Basic information about the subjects in each experiment. The number in parenthesis in the first row indicates the number of subjects that contributed to the analyses (see **Methods**).

	Experiment 1	Experiment 2
Number of subjects	9 (7)	10 (9)
Number tested at 3 months	7	0
Age (range)	18-22	18-22
Age (mean±SD)	20.0±1.4	20.5±1.4

Table S2: Description of content annotations

Two of the authors (A.M. and P.M.) annotated the content of the video clips in the recognition memory test. These annotations were performed blindly to the behavioral responses of the subjects. Below we provide succinct definitions for these annotations, many of which carry a significant degree of subjective evaluation. We also used objective features derived from a computer vision model in **Fig. 5**.

Content	Description	Figure
Faces/scenes	'Faces' indicates presence of a person within the vicinity (~20 ft) of the subject. 'Scenes' includes clips without any other person or situations when there were people in the background. The two labels are mutually exclusive.	3A
Gender	For those clips that contain faces, this annotation indicates whether a male was present and whether a female was present. These definitions are not mutually exclusive (the same clip could contain both). In **Fig. 4A** (Experiment II, target clips), fixation refers to the subset of these clips where the subject fixated on a male or female.	3B, 4A
# Faces	Clips within the faces group that contain either one person or more than one person. The two labels are mutually exclusive. In **Fig. 4B**, fixation refers to the subset of these clips where the subject fixated on one or more people in the clip.	3C, 4B
Age	Subjective estimation of the age of people present in the 'face clips', either younger than the subject or older than the subject. The two labels are not mutually exclusive (there could be both younger and older people in the clip). In **Fig. 4C**, fixation indicates that the subject fixated on people from the corresponding age group.	3D, 4C
Action	Action implies any movement by the person present in the clip other than walking or sitting (e.g. opening a door). No action includes	3E, 4D

	standing, walking or sitting. The two labels are mutually exclusive. In **Fig. 4D**, fixation indicates that the subject fixated on a person executing the action.	
Talking	Talking includes clips where the people (other than the subject) were conversing with each other or talking on the phone. The two labels are mutually exclusive.	3F, 4E
Distinctiveness, faces	'Distinctive' captures the subjective assessment of whether there was anything unusual about the person or people in the clip. A person might stand out because of his actions, looks, attire, etc. The two labels are not mutually exclusive.	3G, 4F
Distinctiveness, objects	Non-distinct objects include doors, chairs, smaller pieces of art placed together in a glass case, etc. Distinct objects include unusual sculptures, objects, etc. Distance may affect whether a smaller sized piece of art is labeled as distinct or not (e.g. a small but intricate vase may be labeled non-distinct when viewed from afar but will be labeled distinct when it is closer to the subject with its intricacies noticeable in the one-second clip). The two labels are not mutually exclusive.	3H, 4G
Sculpture/painting	Whether the clip contained a sculpture or a painting. These are not mutually exclusive annotations.	4H

Brainard D (1997) The Psychophysics Toolbox. Spatial Vision 10:433-436.

Dede AJ, Frascino JC, Wixted JT, Squire LR (2016) Learning and remembering real-world events after medial temporal lobe damage. Proc Natl Acad Sci U S A 113:13480-13485.

Krizhevsky A, Sutskever I, Hinton G (2012) ImageNet Classification with Deep Convolutional Neural Networks. In: NIPS. Montreal.

Peterson MF, Lin J, Zaun I, Kanwisher N (2016) Individual differences in face-looking behavior generalize from the lab to the world. J Vis 16:12.

St Jacques PL, Schacter DL (2013) Modifying memory: selectively enhancing and updating personal memories for a museum tour by reactivating them. Psychol Sci 24:537-543.

Tang H, Singer J, Ison M, Pivazyan G, Romaine M, Frias R, Meller E, Boulin A, Carroll JD, Perron V, Dowcett S, Arlellano M, Kreiman G (2016) Predicting episodic memory formation for movie events. Scientific Reports 6:30175.

Figure S1

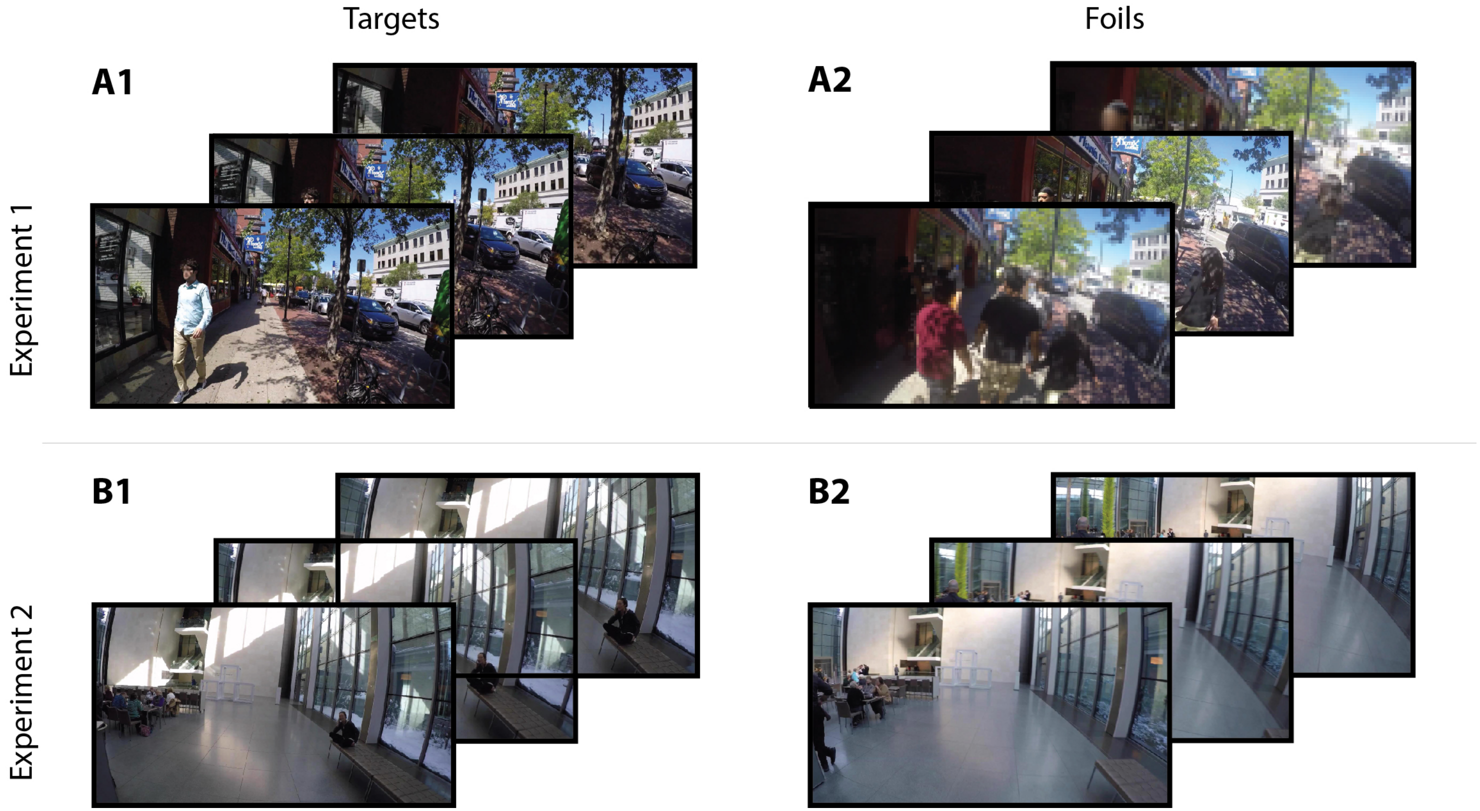

Figure S1. Example target and foil frames. Two example target clips (**A1, B1**) and two example foil clips (**A2, B2**) from Experiment 1 (**A**) and Experiment 2 (**B**). For each video clip, the figure depicts 3 frames (frame 1, 11 and 21) from the sequence of 30 frames presented over 1 second. In each trial, subjects were presented with a single 1-second video clip and had to indicate OLD/NEW (**Methods**); subjects did not have to directly discriminate between the clip in **A1** versus the one in **A2**. These clips are shown alongside here only for comparison purposes. In this rendering (but not in the actual experiment), all faces were blurred due to copyright issues .

Figure S2

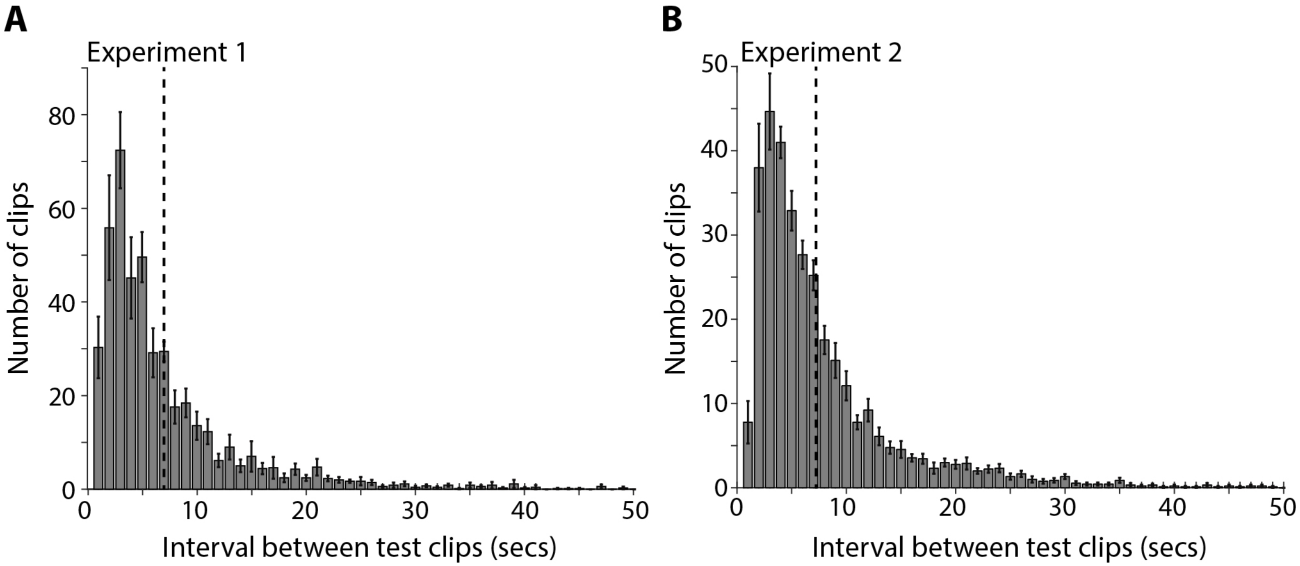

Figure S2. Interval between test clips. Distribution of interval between test clips for Experiment 1 (**A**) and Experiment 2 (**B**). The vertical dashed line marks the average interval.

Figure S3

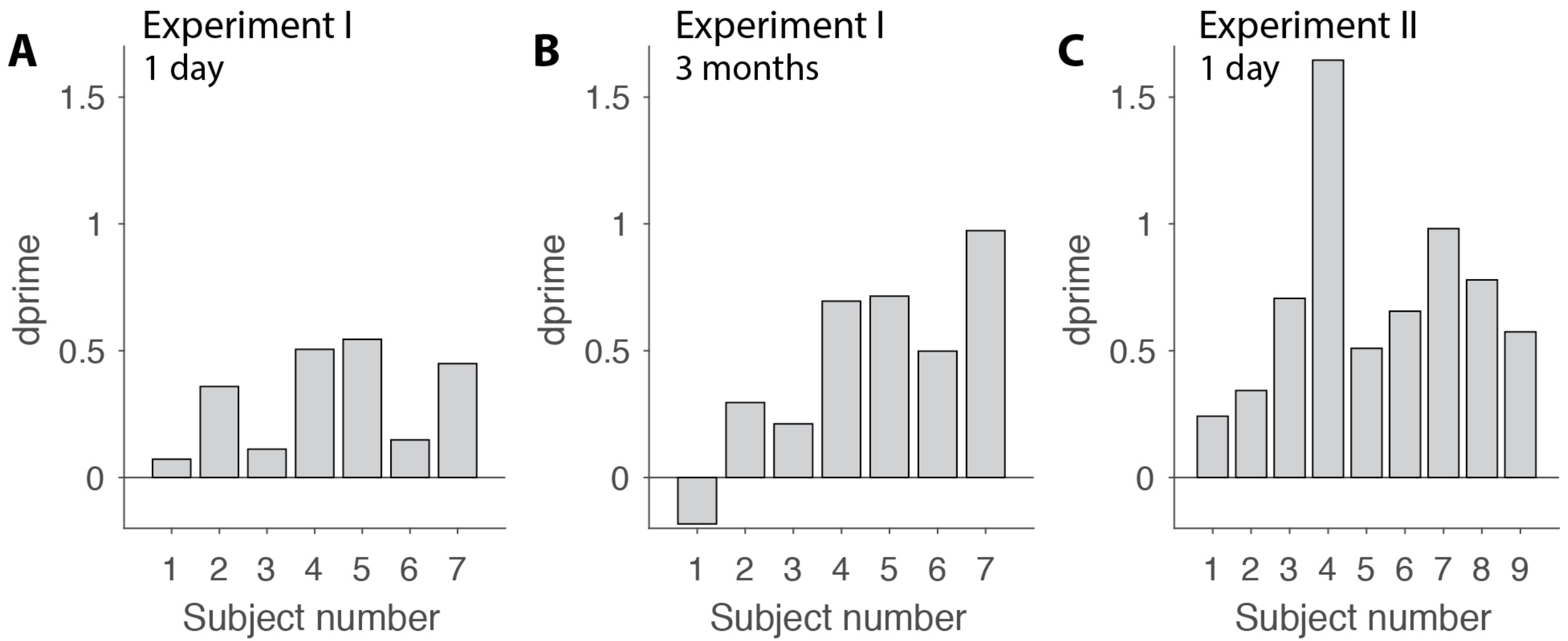

Figure S3. Real life memory performance (d'). Extending **Figure 2A-C**, here we show the d' discrimination metric for each subject. Average values were 0.31±0.2, 0.46±0.38, and 0.72±0.41 (mean±SD) for **A**, **B**, and **C**, respectively.

Figure S4

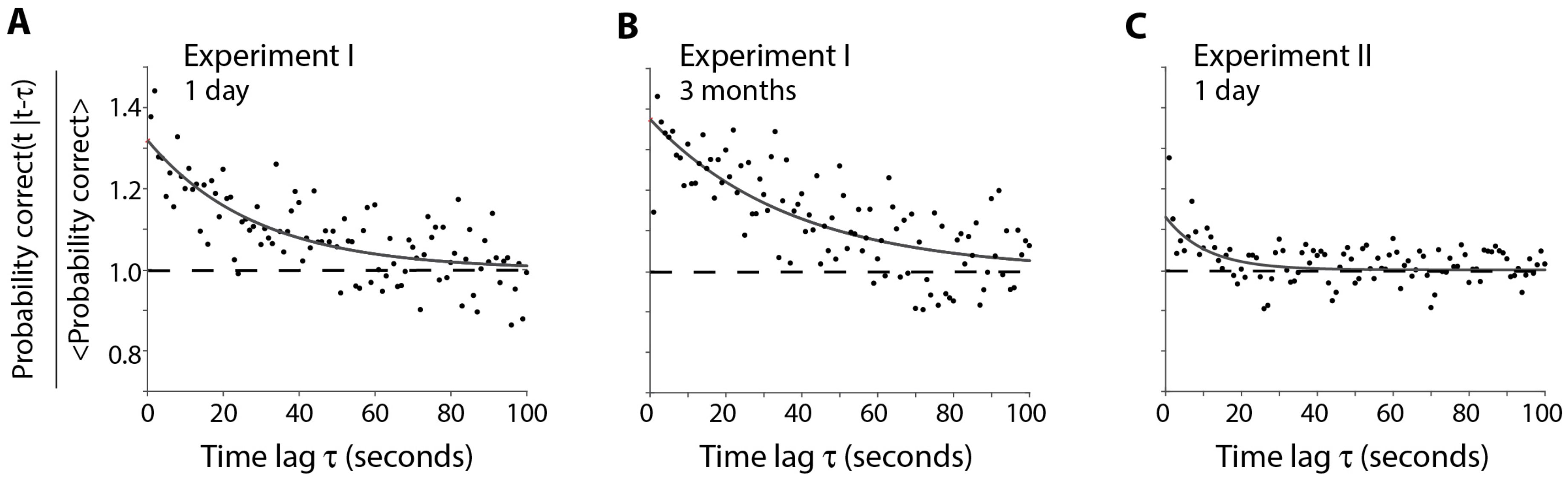

Figure S4. Real life memories show a temporal scale of tens of seconds. History dependence in episodic memory formation during encoding for **A**. Experiment I 1 day test, **B**. Experiment I, 3 months test, and **C**. Experiment II 1 day test. The y-axis indicates the probability that the subject was correct at time t given correct performance at time t-τ, normalized by the average probability of being correct. Results are averaged across subjects. The horizontal dashed line shows the expected value of 1 under the null hypothesis of temporal independence (no history dependence). The solid line shows an exponential fit to the data with time constants 28.4, 37.6 and 10.8 seconds for **A**, **B** and **C** respectively.

Figure S5

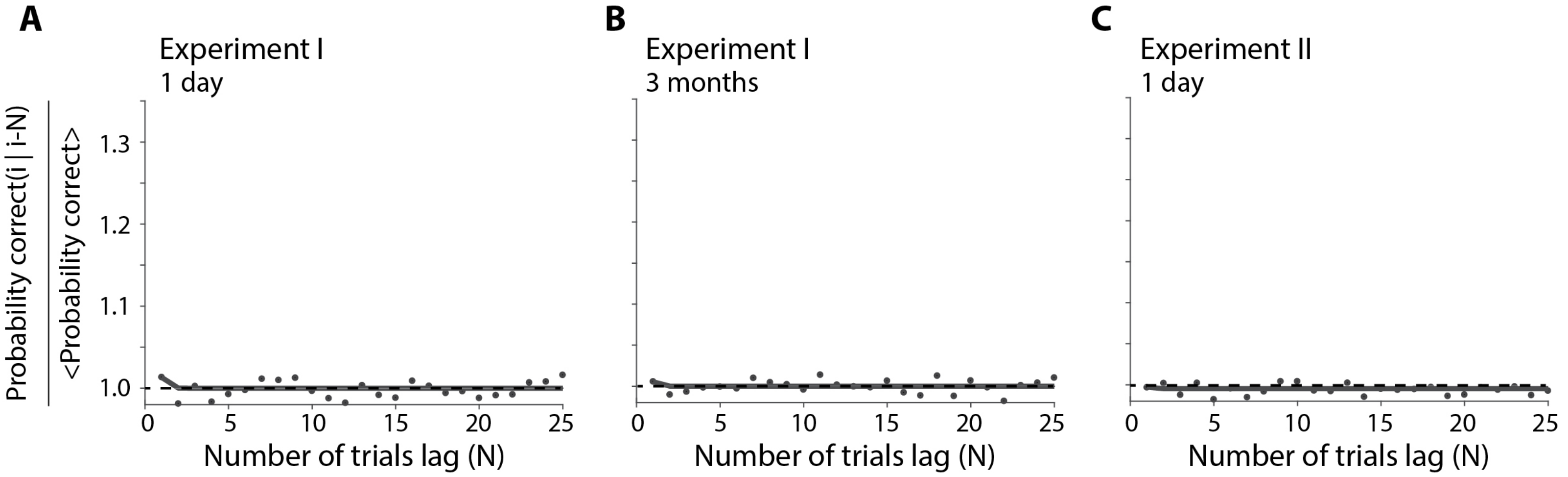

Figure S5. No history dependence during the recognition memory test
This plot is similar to **Figure S4**, except that this is based on the recognition memory test whereas **Figure S4** reports the results during the encoding portion of the experiment. History dependence during recognition memory tests for **A**. Experiment I 1 day test, **B**. Experiment I, 3 months test, and **C**. Experiment II 1 day test. The y-axis indicates the probability that the subject was correct in trial *i* given correct performance at trial *i-N* normalized by the average performance. Results are averaged across subjects. The horizontal dashed line shows the expected value of 1 under the null hypothesis of temporal independence (this line is hard to see because it is behind the exponential fit). The solid line shows an exponential fit to the data with constants -5, -4.7 and -3.9 for **A**, **B** and **C**, respectively.

Figure S6

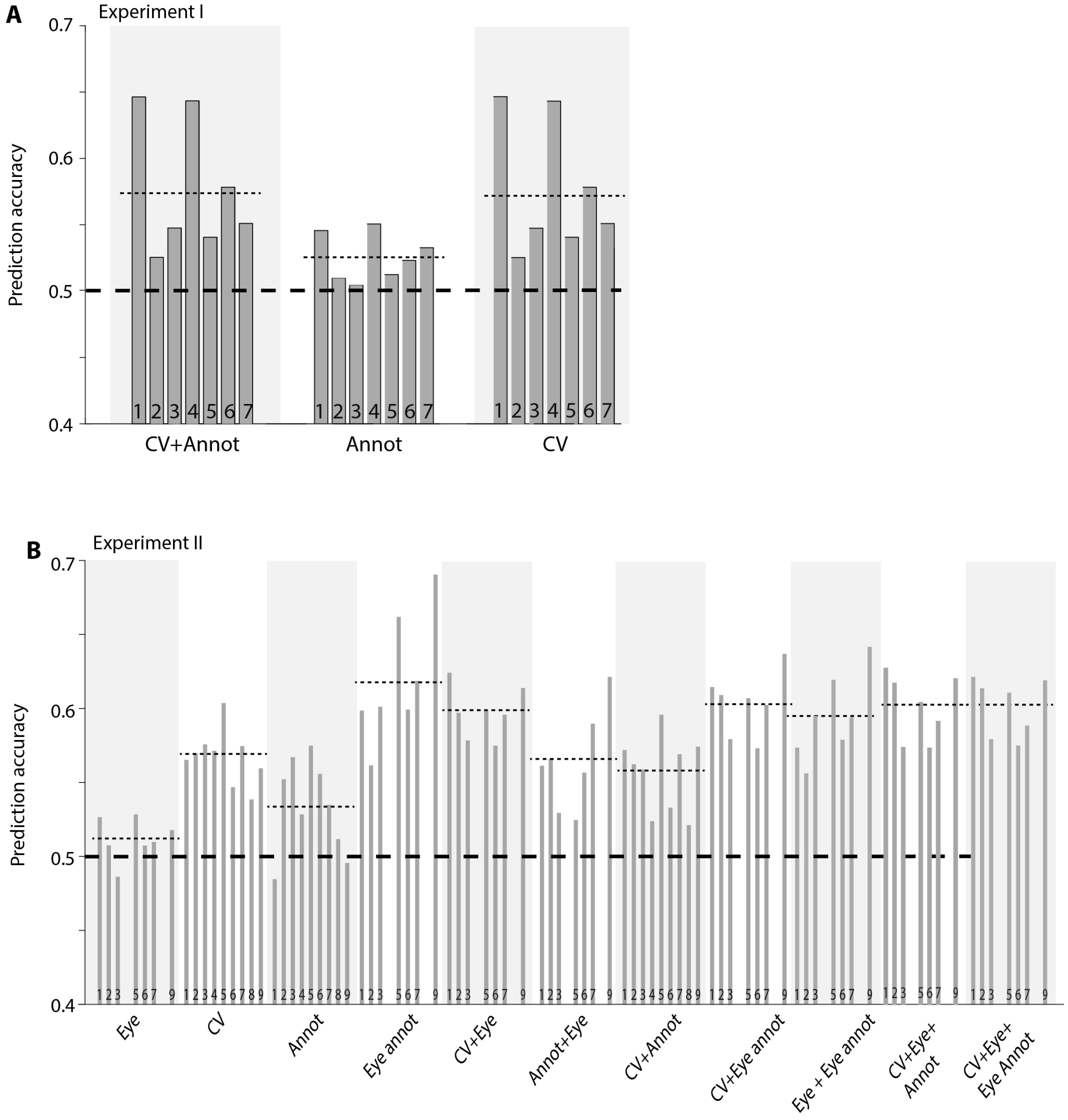

Figure S6. Machine learning prediction of subject performance, individual subjects. Expanding on **Figure 5** in the main text, here we show prediction accuracy for each individual subject for Experiment I (**A**) and Experiment II (**B**) using different types of features: CV = computer vision, Annot = manual content annotations from video, Eye = eye tracking information, Eye annot = annotations of content from eye fixation data (Supplementary Material). The number within each bar denotes the subject number. The thick horizontal dashed line at accuracy = 0.5 denotes chance performance. The thin dotted lines for each type of feature show the average prediction accuracy across subjects. The shaded rectangles are shown only to help visually distinguish the different features used for the classifier. For two subjects (subjects 4 and 8), we could not record accurate eye tracking data and therefore the corresponding classifiers are not shown here.

Figure S7

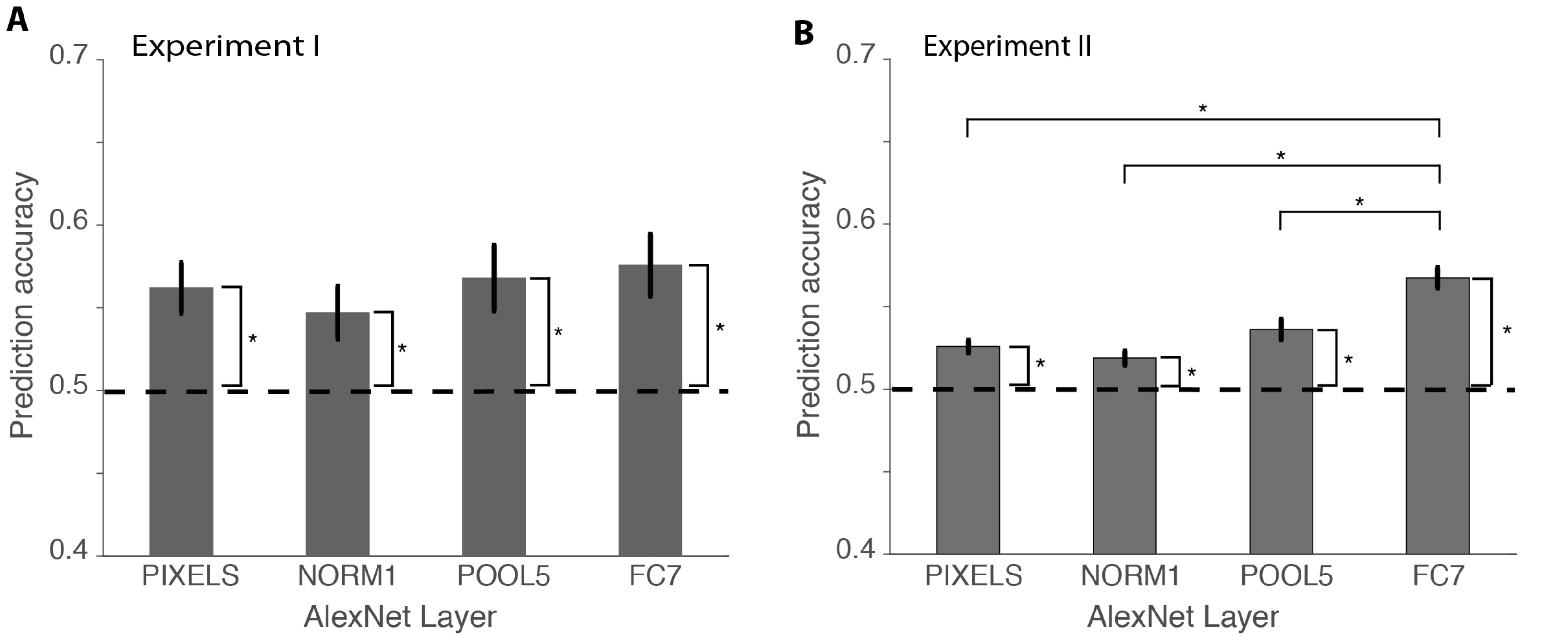

Figure S7. Machine learning prediction of subject performance using different computer vision features
Extending **Figure 5**, this plot shows the prediction accuracy for Experiment I (**A**) and Experiment II (**B**) using different types of features. Norm1, pool5 and fc7 refer to different layers in the AlexNet architecture (see text for details). Error bars = SEM. * denotes statistical significance (p<0.01, ranksum).

Figure S8

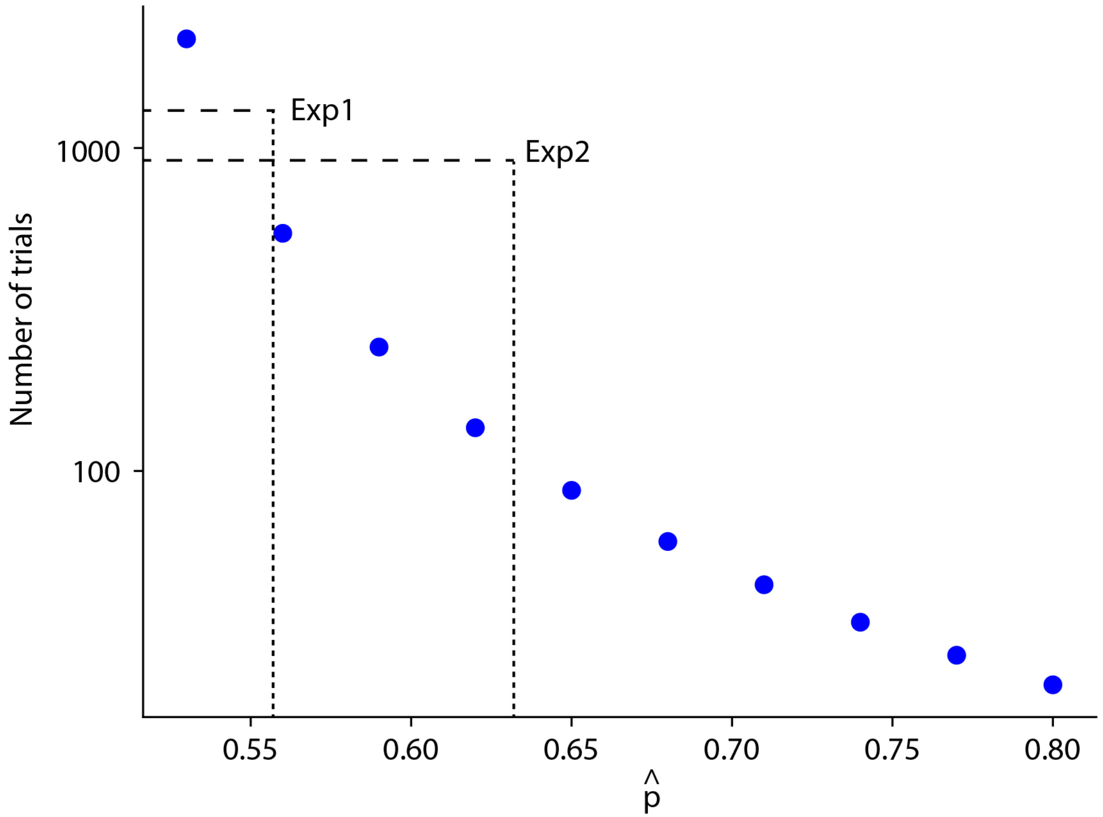

Figure S8. Post-hoc power calculation. Number of trials required to be able to detect a proportion correct > 0.5 assuming that the true proportion correct indicated in the x-axis with a type I error < 0.05 and a power of 0.80. The calculations are based on a conservative estimate of the standard error: $s.e. = \frac{0.5}{\sqrt{n}}$ and solving for *n* in:

$$0.5 + 1.96 s.e. = (p - 0.5) - 0.84 s.e.$$

The horizontal dashed lines indicate the number of trials per subject in Experiments 1 and 2. The vertical dotted lines indicate the average performance reported in Experiments 1 and 2.
